# Palmitate induced secretion of IL-6 and MCP-1 in orbital fibroblasts derived from patients with thyroid-associated ophthalmopathy

**Published:** 2012-06-04

**Authors:** Ji-Sun Paik, Won-Kyung Cho, Eun-Hye Oh, Seong-Beom Lee, Suk-Woo Yang

**Affiliations:** 1 Department of Ophthalmology and Visual Science, Seoul St. Mary’s Hospital, College of Medicine, The Catholic University of Korea, Seoul, Korea; 2 Institute of Hansen’s Disease, College of Medicine, The Catholic University of Korea, Seoul, Korea; 3 Department of Pathology, College of Medicine, The Catholic University of Korea, Seoul, Korea

## Abstract

**Purpose:**

Orbital fibroblasts are now recognized as the key effectors in the development of thyroid associated ophthalmopathy (TAO). TAO is clinically apparent in approximately 50% of patients with Graves’ hyperthyroidism. High levels of plasma free fatty acids (FFAs) are frequently seen in patients with hyperthyroidism. Palmitate is one of the most abundant FFAs in plasma and aggravates inflammation by promoting secretion of pro-inflammatory cytokines in various cells. In the present study, we characterized orbital fibroblasts from patients with TAO and then examined the effect of palmitate on the production of pro-inflammatory cytokines and hyaluronic acid (HA) in orbital fibroblasts.

**Methods:**

Orbital fat explants were obtained from patients with TAO undergoing orbital decompression surgery (n=5). The fibroblasts were characterized by antibodies specific for fibroblast markers and Thy-1 (cluster differentiation 90, CD90) by immunostaining and flow cytometry. We then investigated the capability of orbital fibroblasts to secrete cytokines and HA in response to interleukin (IL)-1β using an enzyme-linked immunosorbent assay (ELISA). The effect of palmitate on cytokine and HA production in orbital fibroblasts was examined at the protein level by ELISA and at the mRNA level by quantitative real time RT–PCR. The level of phosphorylation of mitogen-activated protein kinase (MAPK)s, including p38 MAPK (p38), extracellular signal-regulated kinase (ERK), and c-Jun N-terminal kinase (JNK), was measured by immunoblot analysis. We then examined the role of MAPKs on palmitate-induced cytokine production using specific inhibitors to p38, ERK, and JNK, respectively.

**Results:**

The orbital fibroblasts from patients with TAO were Thy-1- positive fibroblasts (>90%) with the ability to secrete IL-6, IL-8, monocyte chemotactic protein-1 (MCP-1), and HA in response to IL-1β. Treatment with palmitate induced significant production of IL-6 and MCP-1, but not IL-8 and HA, in orbital fibroblasts. IL-6 and MCP-1 expression by palmitate were differentially regulated by MAPKs. IL-6 expression was mediated by the p38, ERK, JNK pathways, whereas MCP-1 expression was mediated by ERK and JNK, but not by p38, in palmitate-treated orbital fibroblasts.

**Conclusions:**

We show the possible involvement of palmitate in the promotion of inflammation within orbital tissues. This finding may be helpful for understanding the development of TAO in patients with hyperthyroidism.

## Introduction

Thyroid associated ophthalmopathy (TAO) is an autoimmune disease affecting orbital and periorbital tissues. The main clinical features of TAO, including upper eyelid retraction, edema, and erythema of the periorbital tissues and conjunctivae, as well as exophthalmos, are mainly due to swelling of the fatty and muscular orbital tissues [1]. The edematous changes that occur in TAO orbital tissues are caused by infiltration of inflammatory cells, accumulation of extracellular matrix (ECM) proteins, proliferation of fibroblasts, and an increased amount of fatty tissue [2].

Orbital fibroblasts are now recognized as the key effectors in the development of TAO and contribute to the development of TAO in several aspects. Orbital fibroblasts are not only main target cells for auto-antibodies present in patients with Graves’ ophthalmopathy but are also involved in inflammation by producing inflammatory cytokines and hyaluronic acid (HA). Thus, many scientists have been interested in factors triggering orbital fibroblasts to secrete pro-inflammatory cytokines. In addition to autoantibodies, ganglioside [3], and cluster differentiation 154 (CD154), the CD40 cognate ligand [4], induces secretion of pro-inflammatory cytokines from orbital fibroblasts.

TAO is associated with hyperthyroidism, although it may occur in hypothyroid or euthyroid patients. TAO is clinically apparent in approximately 50% of patients with Graves’ hyperthyroidism [5]. Glucose intolerance and high levels of plasma free fatty acids (FFAs) are frequently seen in patients with hyperthyroidism, and these may be caused by the hypermetabolic state due to elevated serum thyroid hormones [6]. Elevated plasma FFAs are associated with insulin resistance in skeletal muscle [7] and endothelial dysfunction in the cardiovascular system [8]. Of various serum FFAs, palmitate (C16:0) has received the most attention for its ability to induce cardiomyocyte cell death [9]. In addition to cardiac toxicity, palmitate not only inhibits insulin signaling in skeletal muscle cells [10] and induces cell death in pancreatic β-cells [11], it also aggravates inflammation by promoting secretion of pro-inflammatory cytokines in various cells [12-16]. Thus, we thought that palmitate may also induce the secretion of pro-inflammatory cytokines from orbital fibroblasts, although there is not yet scientific evidence that supports the correlation between plasma FFA levels and the development of TAO.

In this study, we examined the possible involvement of FFAs, particularly palmitate, in the promotion of inflammation within orbital tissues and in the subsequent development of TAO. We initially characterized orbital fibroblasts from patients with TAO. We assessed the effect of palmitate on the production of pro-inflammatory cytokines and HA in orbital fibroblasts.

## Methods

### Reagents and antibodies

Palmitate, fumonisin B1, and triacsin C were obtained from Sigma-Aldrich Co. Ltd (St. Louis, MO). The inhibitors, SB 203580 (p38 MAPK [p38]), PD 98059 (MAPK kinase 1 [MEK1]), and SP 600125 (c-Jun N-terminal kinase [JNK]/ stress activated protein kinase [SAPK]) were purchased from Calbiochem (La Jolla, CA). Fumonisin B1, triacsin C, SB 203580, PD 98059, and SP 600125 were dissolved in dimethyl sulfoxide or methyl alcohol or H_2_O. The final vehicle concentration was adjusted to 0.1% (v/v), and the control medium contained the same quantity of vehicle. Antibodies against phospho-p38, p38, phospho-ERK, ERK, phospho-JNK, JNK, and PDI (disulfide isomerase) were obtained from Cell Signaling Technology (Beverly, MA). Antibodies against vimentin, α-smooth muscle actin, and cytokeratin were purchased from Abcam (Cambridge, UK). Antibody against factor VIII was obtained from Sigma-Aldrich. PE-Cy5-conjugated anti-CD90 (Thy-1) antibody and PE-Cy5-conjugated immunoglobulin G kappa (IgGĸ) isotype were obtained from BD PharMingen (San Jose, CA). anti-Glyceraldehyde-3-phosphate-dehydogenase (anti-GAPDH) antibody, horseradish peroxidase, and Cy3-conjugated secondary antibodies were obtained from Santa Cruz Biotechnology (Santa Cruz, CA).

### Establishment of orbital fat-derived fibroblasts

Orbital fat explants were obtained as surgical waste during orbital bone and fat decompression surgery in patients with TAO (n=5). Before the decompression surgery, all patients with TAO had experienced at least 6 months of inactive disease status with a euthyroid condition. Patient characteristics are presented in Table 1. Orbital fat explants were chopped in small pieces, attached to plastic culture dishes, and covered with Dulbecco’s Modified Eagle’s medium (GIBCO BRL, Grand Island, NY) supplemented with 20 mM HEPES (Fisher Scientific, Atlanta, GA), 10% fetal bovine serum (FBS; GIBCO BRL), 100 U/ml of penicillin, and 100 μg/ml of streptomycin (Bio Whittaker Inc., Walkersville, MD). Dermal fibroblasts were obtained from skin specimens of healthy patients without TAO undergoing abdomen reduction surgery. Cultures were maintained at 37 °C in a 5% CO_2_ humidified incubator until fibroblasts reached 70% confluence. Non-adherent cells and fat tissues were then removed, and the established fibroblasts were passaged with gentle trypsin/EDTA treatment. Fibroblasts were not used for studies beyond passage 7 from the initial culture. These activities were undertaken after informed consent was obtained from the donors, according to procedures approved by the Institutional Review Board of Seoul St. Mary’s Hospital (KC10TISE0743) and the tenets of the Declaration of Helsinki.

**Table 1:** Clinical characteristics of patients with thyroid associated ophthalmopathy at time of orbital decompression surgery.

** **	** **	** **	**Graves’ disease**			**Treatment TAO**			** **	** **	** **
**Patient**	**Age/sex**	**Smoker**	**Radioiodine therapy**	**Surgery**	**Metimazole**	**Surgery**	**Prednisone**	**Radiation**	**Euthyroid**	**TSH-rAb**	CAS*
#1	F/38	No	No	No	Yes	Yes	No	No	Yes	Yes	0
#2	M/54	No	No	No	Yes	Yes	Yes	Yes	Yes	Yes	2
#3	M/47	No	No	No	Yes	Yes	No	Yes	Yes	Yes	1
#4	F/39	No	No	No	Yes	Yes	Yes	No	Yes	Yes	1
#5	M/48	No	No	No	Yes	Yes	No	No	Yes	Yes	1

*CAS-Clinical activity score.

### Characterization of orbital fat-derived fibroblasts

Fibroblasts were characterized by immunostaining and flow cytometry. The expression patterns of fibroblast and non-fibroblast markers were examined by immunostaining with antibodies against vimentin, α-smooth muscle actin, PDI, factor VIII, and cytokeratin. Cells were fixed in 2% paraformaldehyde in PBS and permeabilized by 5% Triton X-100 in PBS. Fixed cells were rinsed with PBS and incubated in blocking solution (0.1 M NH_4_Cl, 0.2% gelatin, 0.3% Triton X-100 in PBS) for 20 min. The cells were then incubated overnight with a primary antibody against the designated protein in an incubation solution (0.2% gelatin, 0.3% Triton X-100, and 3% goat serum in PBS) at 4 °C. After washing with PBS, cells were incubated with the corresponding Cy3-conjugated secondary IgG at room temperature for 2 h. Nuclei were counterstained for 15 min with 4'-6-diamidino-2-phenylindole (Sigma). The negative control was processed without primary antibody. Immunofluorescence was visualized by inverted fluorescence microscopy (IX71/IX51; Olympus Corp., Tokyo, Japan). The Thy-1 CD90 expression pattern was examined by flow cytometry with an antibody against Thy-1. Cells were suspended in FACS buffer (0.05% NaN_3_, 5% FBS, 95% PBS) and then incubated with PE-Cy5-conjugated anti-Thy-1 antibody. PE-Cy5-conjugated IgG1 κ isotype was used as a negative control. The cells were washed twice with FACS buffer, resuspended, and analyzed by flow cytometry (FACSCalibur, BD Bioscience).

### Palmitate preparation

Palmitate was prepared, as described previously [17]. In brief, to reduce endotoxin contamination, palmitate was dissolved to a final concentration of 40 mM in a solvent of 0.1 N NaOH/70% ethanol and added directly to the cell culture media, where it could complex with BSA already present in the culture media. Palmitate was used at final concentrations of 100–400 μ.

### Endotoxin assay

The level of endotoxin was measured using a chromogenic endotoxin assay kit (the ToxinSensor^TM^ Endotoxin Detection System; GenScript, Piscataway, NJ). Briefly, reconstituted limulus amebocytes lysates was mixed with sample solution and the mixture was incubated at 37 °C for 45 min. After incubation, chromogenic substrate solution was added to the mixture and incubated at 37 °C for 6 min. Color stabilizer was then added and the absorbance of each reaction was read at 545 nm.

### Cytokine assays

Fibroblasts were cultured to confluence and treated for 1 h with or without a designated inhibitor, followed by treatment with palmitate (100–400 μM) for 24 h. Culture medium was analyzed for interleukin (IL)-6, IL-8, and monocyte chemotactic protein-1 (MCP-1) content using a standard sandwich enzyme linked immunosorbent assay (ELISA) kit (R&D Systems, Minneapolis, MN). HA concentration was determined with an HA-ELISA kit (Echelon Biosciences, Salt Lake City, UT) according to the manufacturer’s instructions.

### Immunoblot analysis

Treated cells were removed from the incubator at the designated time, placed on ice, and washed three times with ice-cold PBS. The cells were then lysed for 30 min with RIPA lysis buffer (50 mM Tris-HCl [pH 7.4], 1% Triton X-100, 150 mM NaCl, 0.1% SDS, 0.5% sodium deoxycholate, 100 mM phenylmethylsulfonyl fluoride, 1 μg/ml of leupeptin, 1 mM Na_3_VO_4_, and 1× Complete ^TM^ Protease Inhibitor Cocktail; Santa Cruz Biotechnology). Equal amounts of protein were loaded onto 10%–15% SDS–PAGE gels, electrophoresed, and transferred onto PVDF membranes (Millipore, Bedford, MA). The membranes were blocked in Tris-buffered saline with 0.05% Tween-20 (TBST) supplemented with 5% powdered milk or 5% BSA and then incubated with a primary antibody against the designated protein. The blot was then washed with TBST and incubated with a horseradish peroxidase-conjugated secondary antibody in TBST plus 5% powdered milk. The bound antibodies were detected with Super Signal Ultra Chemiluminescence Reagents (Pierce Biotechnology, Inc., Rockford, IL).

### Quantitative Real Time-RT–PCR

*IL-6* and *MCP-1* mRNA expression was determined by quantitative real time-RT–PCR. Briefly, total RNA was extracted using a High Pure RNA Isolation kit (Roche Diagnostics, Mannheim, Germany) and converted to cDNA using an Advantage RT-for-PCR kit (Clontech, Hampshire, UK), according to the manufacturer’s instructions. To quantify *IL-6* and *MCP-1* mRNA, quantitative real time RT–PCR was performed with a iQ^TM^ SYBR**^®^** Supermix kit (Bio-Rad Laboratories, Hercules, CA) in a Peltier Thermal Cycler-200 system (MJ Research, Berlin, Germany). Real time PCR was performed in triplicate at 95 °C for 3 min followed by 35 cycles of amplification (94 °C for 30 s, 68.3 °C for 30 s, and 72 °C for 30 s). The relative amounts of *IL-6* and *MCP-1* mRNA were determined by subtracting the cycle threshold (Ct) values from the Ct values for cyclophilin. The following primers for *IL-6*, *MCP-1*, and cyclophilin were used: For *IL-6*, forward primer (5′-AAA TGC CAG CCT GCT GAC GAA C-3′) and reverse primer (5′-AAC AAC AAT CTG AGG TGC CCA TGC TAC-3′). For *MCP-1*, forward primer (5′-ATG CAA TCA ATG CCC CAG TC-3′) and reverse primer (5′-TGC AGA TTC TTG GGT TGT GG-3′). For cyclophilin, forward primer (5′-GCA TAC GGG TCC TGG CAT CTT GTC C-3′) and reverse primer (5′-ATG GTG ATC TTC TTG CTG GTC TTG-3′).

### Statistical analyses

All results are expressed as mean±SD. An unpaired Student’s *t*-test was used to determine differences from control values. Statistical software (SPSS 15.0; SPSS inc., Chicago, IL) was used for analysis, and p<0.05 was considered statistically significant.

## Results

### Characterization of orbital fibroblasts from patients with TAO

**Figure 1:**
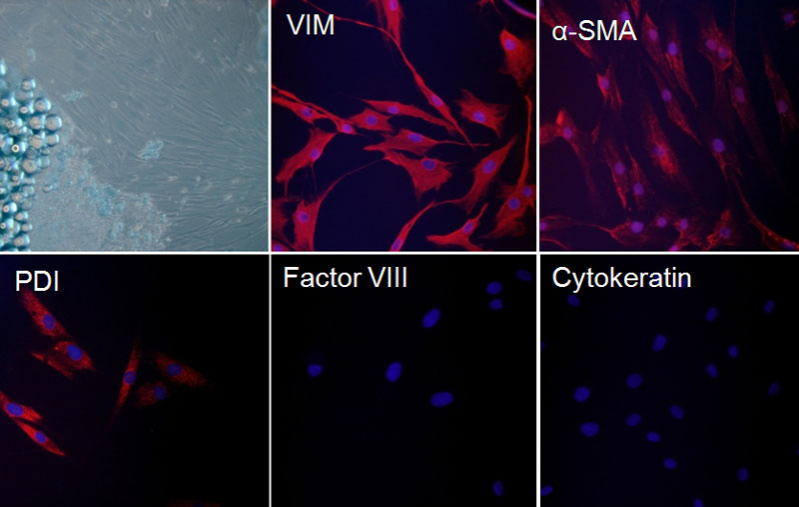
Characterization of orbital fibroblasts. Orbital fibroblasts were derived from retrobulbar fat tissue of patients with thyroid-associated ophthalmopathy (n=5, Table 1). Morphology was examined by phase-contrast microscopy and the expression patterns of fibroblast and non-fibroblast markers were examined by immunostaining with antibodies against vimentin (VIM), α-smooth muscle actin (α-SMA), protein disulfide-isomerase (PDI), factor VIII, and cytokeratin, respectively. Images were obtained from orbital fibroblasts of a representative patient with TAO. Similar results were observed in orbital fibroblasts from all five patients with TAO.

Orbital fibroblasts, derived from orbital fat explants of patients with TAO, were flat and elongated with processes extending out from the ends of the cell bodies. We initially examined the expression of fibroblast markers in orbital fibroblasts by immunostaining to characterize the orbital fibroblast phenotypes. As shown in Figure 1, the orbital fibroblasts stained positively for vimentin (a fibroblast marker), α-smooth muscle actin (a myofibroblast marker), and protein disulfide-isomerase (a fibroblast marker) but negatively for factor VIII (an endothelial cell marker) and cytokeratin (a keratinocyte marker).

**Figure 2:**
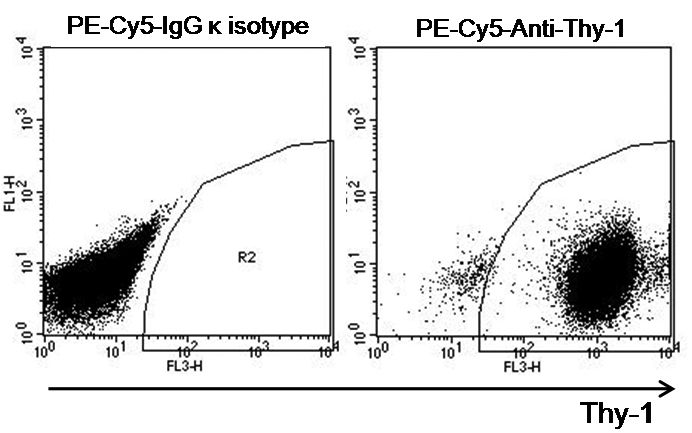
Flow cytometry analysis of Thy-1 expression in orbital fibroblasts. Orbital fat-derived fibroblasts were stained with a PE-Cy5-IgG κ isotype and a PE-Cy5-anti-Thy-1 antibody, respectively, and then analyzed by a flow cytometry. Data are obtained from orbital fibroblasts of a representative patient with TAO.

Orbital fibroblasts are heterogeneous with respect to surface Thy-1 expression. Thy-1-positive and negative subpopulations play distinct roles in the development of TAO. Therefore, we analyzed the proportion of Thy-1-positive and negative cells by flow cytometry. Expression of Thy-1 in fibroblasts derived from orbital fat explants of patients with TAO in our system was relatively homogeneous and >90% (Figure 2).

**Figure 3:**
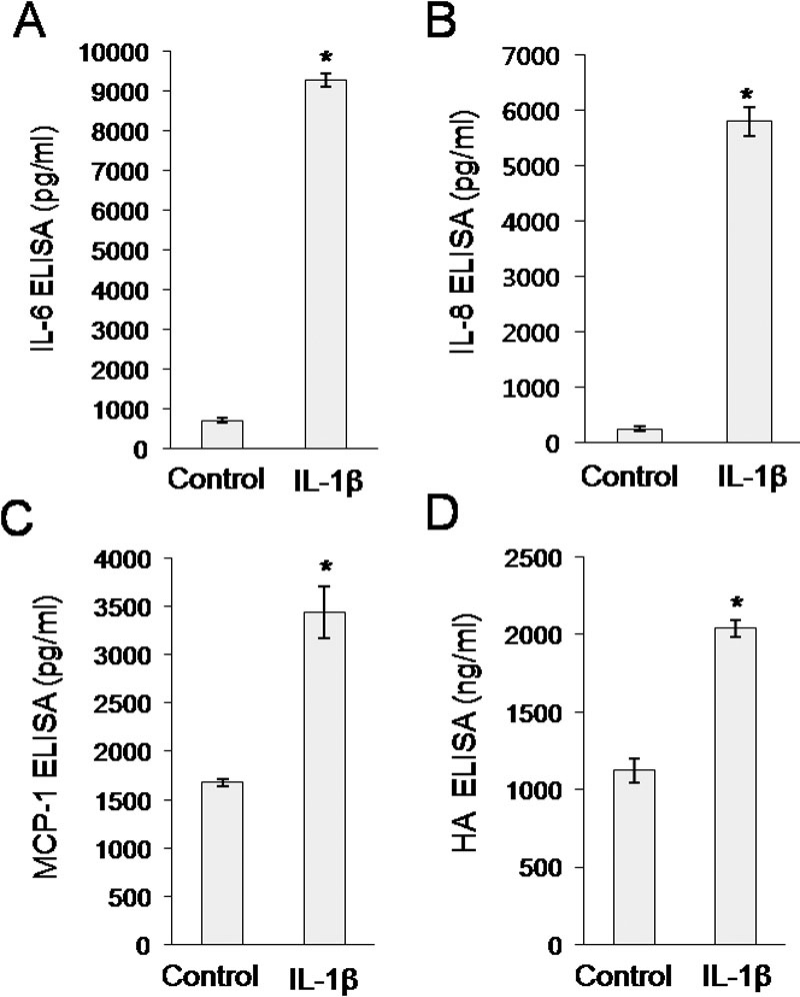
Secretion of IL-6, IL-8, MCP-1, and hyaluronic acid (HA) from orbital fibroblasts in response to IL-1β. Orbital fat-derived fibroblasts were treated with IL-1β (10 ng/ml) for 24 h. Concentrations of IL-6 (A), IL-8 (B), MCP-1 (C), and HA (D) were determined by ELISA. Data are presented as the mean±SD of three replicate culture wells from a representative experiment. Similar results were observed in orbital fibroblasts from #1 and #2 patients with TAO (Table 1). * p<0.05.

We next investigated the capability of orbital fibroblasts to secrete cytokines and HA. Orbital fibroblasts were treated with 10 ng/ml IL-1β for 24 h. Treatment with IL-1β induced secretion of IL-6, IL-8, MCP-1, and HA by orbital fibroblasts. The level of secretion of IL-6, IL-8, MCP-1 and HA increased 13.0 fold, 22.7 fold, 2.0 fold, and 1.8 fold, respectively, compared with control levels (Figure 3). These results show that orbital fibroblasts are Thy-1-positive fibroblasts with the ability to secrete IL-6, IL-8, MCP-1, and HA in response to IL-1β.

### Effect of palmitate on IL-6 and MCP-1 secretion in orbital fibroblasts

**Figure 4:**
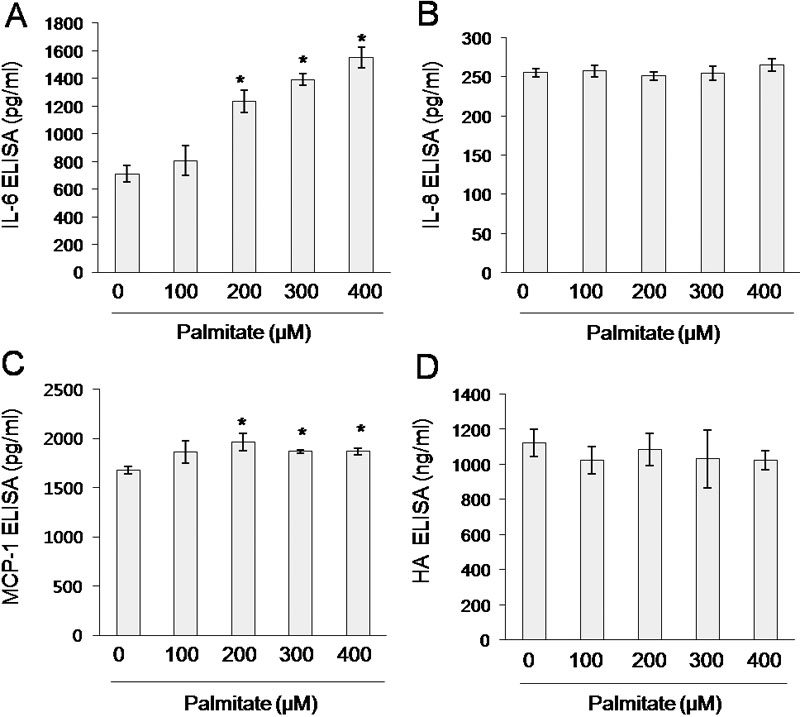
Secretion of IL-6 and MCP-1, but not IL-8 and hyaluronic acid (HA), from orbital fibroblasts in response to palmitate. Orbital fibroblasts were treated with palmitate for 24 h. Concentrations of IL-6 (**A**), IL-8 (**B**), MCP-1 (**C**), and HA (**D**) were determined by ELISA. Data are presented as the mean±SD of three replicate culture wells from a representative experiment. Similar results were observed in three independent experiments using orbital fibroblasts from #1 patient with TAO (Table 1). * p<0.05 versus non-treated control cells.

**Figure 5:**
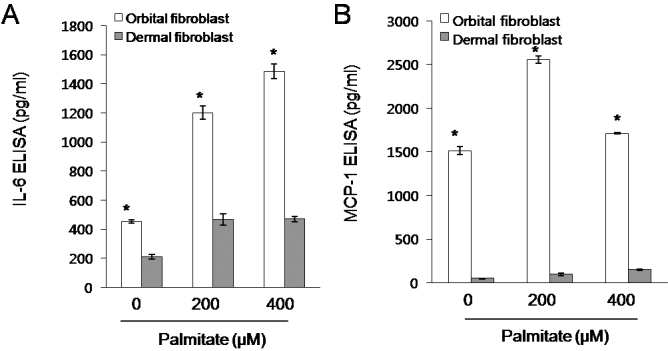
Higher levels of IL-6 and MCP-1 secreted from orbital fibroblasts than from dermal fibroblasts under basal conditions and in response to palmitate. Orbital and dermal fibroblasts were treated with palmitate for 24 h. Concentrations of IL-6 (**A**) and MCP-1 (**B**) were determined by ELISA. Data are presented as the mean±SD of three replicate culture wells from a representative experiment. Similar results were observed in three independent experiments using orbital fibroblasts from #1 patient with TAO (Table 1). * p<0.05 versus cells treated with same concentration of palmitate.

Following this, we examined the effects of palmitate on secretion of IL-6, IL-8, MCP-1, and HA by orbital fibroblasts. Orbital fibroblasts were treated with 100–400 μM palmitate for 24 h. As shown in Figure 4, palmitate treatment significantly induced IL-6 secretion in a dose dependent manner and slightly but significantly induced MCP-1 secretion in orbital fibroblasts. In addition, orbital fibroblasts secreted higher amounts of IL-6 and MCP-1 under basal conditions and in response to palmitate, compared with dermal fibroblasts (Figure 5). However, palmitate did not increase IL-8 or HA secretion in orbital fibroblasts (Figure 4).

**Figure 6:**
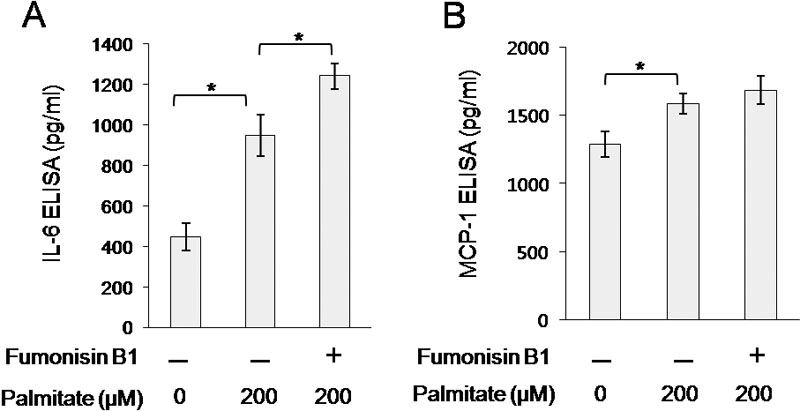
Effect of fumonisin B1 on the palmitate-induced IL-6 and MCP-1 secretion from orbital fibroblasts. Orbital fibroblasts were pretreated with fuminosin B1 (10 μM) for 1 h, followed by treatment with palmitate (200 μM) for 24 h. Concentrations of IL-6 (**A**) and MCP-1 (**B**) were determined by ELISA. Data are presented as the mean±SD of three replicate culture wells from a representative experiment. Similar results were observed in three independent experiments using orbital fibroblasts from #1 patient with TAO (Table 1). * p<0.05.

Palmitate has been implicated in various biologic phenomena via the ceramide pathway. Ceramide is produced from palmitate via a de novo synthetic pathway of serine-palmitoyl transferase [18]. Thus, we examined whether the palmitate-induced secretion of IL-6 and MCP-1 is dependent on de novo ceramide synthesis. Orbital fibroblasts were pretreated with 10 μM fumonisin B1, an inhibitor of de novo ceramide synthesis, for 1 h followed by treatment with palmitate (200 μM) for 24 h. Pre-treatment with fumonisin B1 enhanced rather than inhibited palmitate-induced IL-6 and MCP-1 secretion from orbital fibroblasts (Figure 6). These results show that palmitate induces IL-6 and MCP-1 secretion via a ceramide-independent pathway in orbital fibroblasts.

### Regulation of palmitate-induced IL-6 and MCP-1 expression by MAPKs in orbital fibroblasts

**Figure 7:**
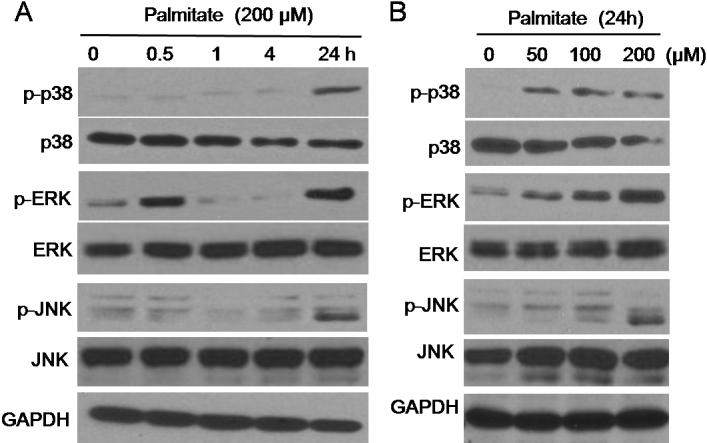
Effect of palmitate on p38, ERK, and JNK phosphorylation in orbital fibroblasts. Orbital fibroblasts were treated with palmitate (200 μM) for 0.5– 24 h (**A**) or treated with palmitate (50–200 μM) for 24 h (**B**). Levels of phosphorylated p38, ERK, and JNK were determined by immunoblot analysis. Data are representative of three independent experiments using orbital fibroblasts from #1, #3, and #4 patients with TAO (Table 1).

Mitogen-activated protein kinases (MAPKs) play a key role in regulating fibroblast cytokine production. Previous studies have shown that IL-1β-induced IL-6 secretion is mediated by p38 and ERK pathways in orbital fibroblasts [19] and that IL-18-induced MCP-1 expression is mediated by JNK in synovial fibroblasts [20]. Thus, we investigated whether palmitate-induced IL-6 and MCP-1 expression in orbital fibroblasts were mediated by p38, ERK, and JNK pathways. We initially measured the levels of phosphorylated p38, ERK, and JNK in palmitate-treated orbital fibroblasts by immunoblot analysis. As shown in Figure 7, treatment with palmitate induced p38, ERK, and JNK phosphorylation in orbital fibroblasts in a time (Figure 7A) and dose (Figure 7B)-dependent manner. Interestingly, ERK phosphorylation by palmitate occurred in dual peaks, at 0.5 and 24 h.

**Figure 8:**
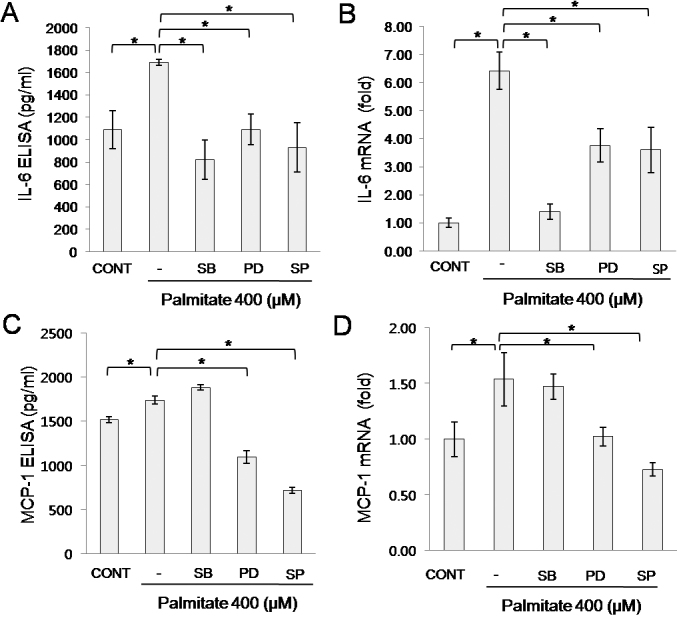
Regulation of palmitate-induced IL-6 and MCP-1 production by mitogen activated protein kinases (MAPKs) in orbital fibroblasts. Orbital fibroblasts were treated for 1 h with or without the designated inhibitor (20 μM), followed by treatment with palmitate (400 μM) for 24 h. Protein concentrations of IL-6 (**A**) and MCP-1 (C) were determined by ELISA. mRNA levels of *IL-6* (**B**) and *MCP-1* (**D**) were determined by quantitative real time-RT–PCR. Data are presented as the mean±SD of triplicate samples from a representative experiment. Similar results were observed in orbital fibroblasts from #1, #3, and #5 patients with TAO (Table 1). *p<0.05. SB, SB203580; PD, PD98059; and SP, SP 600125.

We then examined the role of MAPKs in palmitate-induced IL-6 and MCP-1 secretion in orbital fibroblasts using specific inhibitors, such as SB 203580 for p38, PD98059 for ERK, and SP600125 for JNK. Orbital fibroblasts were treated for 1 h with or without the designated MAPK inhibitor at 20 μM, followed by treatment with palmitate (400 μM) for 24 h. Palmitate-induced IL-6 expression was suppressed by inhibitors of p38 (SB 203580), ERK (PD98059), and JNK (SP 600125) at both the protein and mRNA levels (Figure 8A,B). MCP-1 protein and mRNA expression were also suppressed by inhibitors of ERK (PD98059) and JNK (SP 600125), but not by the p38 inhibitor (SB 203580) in palmitate-treated orbital fibroblasts (Figure 8C,D). These results show that IL-6 and MCP-1 expression by palmitate were differentially regulated by MAPKs. IL-6 expression was mediated by the p38, ERK, JNK pathways, whereas MCP-1 expression was mediated by ERK and JNK, but not by p38, in palmitate-treated orbital fibroblasts.

## Discussion

Orbital fibroblasts are sub-grouped into Thy-1-positive and negative cells. These subsets exhibit distinct phenotypes and contribute differently to the development of TAO. Thy-1-positive cells are capable of myofibroblast differentiation after treatment with transforming growth factor-β and produce more prostaglandin E2 and HA than that of Thy-1- negative cells after stimulation with IL-1β or GT1b, whereas Thy-1-negative cells differentiate into lipofibroblasts after adipogenic stimulation and produce higher levels of IL-8 than that of Thy-1-positive cells after treatment with IFN-γ [4,21,22]. Thus, the clinical manifestation of TAO may vary depending on the relative proportion of Thy-1-positive and negative cells. Khoo et al. [23] reported that the proportion of orbital Thy-1-positive cells increased from 66.8% (range, 63.3%–71.0%) in normal fibroblast cultures to 77.9% (range, 66.5%–84.8%) in cultured orbital fibroblasts from patients with Grave’s ophthalmopathy. In the current study, the proportion of Thy-1-positive cells (90.1±7.0%; range, 85.7%–98.2%), which was derived from orbital fat explants of patients with TAO, in our system was slightly higher, as compared to the level of a previous report (Figure 2) [23].

Here we demonstrated that orbital fibroblasts secreted higher amounts of IL-6 and MCP-1 under basal conditions and in response to palmitate, compared with dermal fibroblasts (Figure 5). It has been reported that palmitate induces the secretion of IL-6 and MCP-1 from various types of cells [12-15]. In addition, Chen et al. [19] reported that orbital fibroblasts, when treated with IL-1β, expressed higher level of IL-6 than dermal firborblasts, suggesting that the capacity of orbital fibroblasts to express pro-inflammatory cytokines may underlie the particular susceptibility of orbital connective tissues to immune activation.

Although Ceramide, a metabolite of palmitate, is a well known inducer of pro-inflammatory cytokines [16], palmitate induces the production of pro-inflammatory cytokines via a ceramide-dependent or independent pathway, depending on the type of palmitate-induced cytokine and the cell. Furthermore, inhibiting ceramide synthesis enhances palmitate-induced secretion of cytokines in certain cells [12]. Pretreatment with triacsin C, an inhibitor of acyl-CoA synthetase in de novo ceramide synthesis, reduces palmitate-induced secretion of tumor necrosis factor (TNF)-α, IL-8, and IL-1β in human macrophages [16], whereas pretreatment augments palmitate-induced IL-6 secretion in adipocytes [12]. Our results also show that pretreatment with fuminosin B1 (Figure 5) or triacsin C (data not shown), inhibitors of de novo ceramide synthesis, increased rather than inhibited IL-6 and MCP-1 secretion from palmitate-treated orbital fibroblasts. The biochemical mechanism of how inhibiting ceramide synthesis enhances secretion of IL-6 and MCP-1 is unclear, but sphinganine, sphingosine, and their 1-phosphate metabolites, which increase when ceramide synthesis is inhibited by fumonisin B1, are likely to participate in this regulation. In particular, sphigosine-1-phosphate appears to have both pro- and anti-inflammatory effects depending on the cell context [24]. In addition, we considered the direct effect of fumonisin B1 on IL-6 and MCP-1 secretion, as treatment of mice with fumonisin B1 increases levels of IL-12, TNF-α, interferon γ, and IL-1 in mouse tissues [25]. However, in our pilot study, treatment with 10 μM fumonisin B1 had no effect on IL-6 or MCP-1 secretion in RAW 264.7 cells (data not shown).

In the context of TAO immunological features, IL-6 and PCP-1 play an important role in lymphocyte function. IL-6 not only drives the synthesis of immunoglobulins in B cells but also induces Th2 differentiation in T cells [26]. Hiromatsu et al. [26] reported that IL-6 mRNA is highly expressed in extra-ocular eye muscles and orbital fat tissue in patients with TAO, and that its level is positively correlated with orbital volume, showing the importance of IL-6 in the pathogenesis of TAO. Additionally, MCP-1 also contributes to the development of TAO by attracting leukocytes. *MCP-1* mRNA is highly expressed in orbital adipose tissues of patients with TAO, compared with that in orbital adipose tissues from healthy donors [27].

Interestingly, the action of p38 on IL-6 and MCP-1 production was different in palmitate-treated orbital fibroblasts, although palmitate induced phosphorylation of all MAPKs such as p38, ERK, and JNK (Figure 6). Pre-treatment with inhibitors of p38 (SB 203580), ERK (PD98059), and JNK (SP 600125) suppressed IL-6 expression in palmitate-treated orbital fibroblasts, whereas MCP-1 was suppressed by inhibitors of ERK (PD98059) and JNK (SP 600125), but not by a p38 inhibitor (SB 203580) (Figure 7C,D). While the exact mechanism of IL-6 and MCP-1 regulation by MAPKs is unknown, there are several reports showing different p38 actions between IL-6 and MCP-1. Kito et al. [28] reported that cyclic strain-induced IL-6 production is regulated by p38, ERK, and JNK in endothelial cells, whereas Amin et al. [20] reported that IL-18-induced MCP-1 production in synovial fibroblasts of patients with rheumatoid arthritis is mediated by JNK, but not p38.

Plasma FFAs are used as an oxidative energy source and as a signaling molecule in various tissues such as skeletal muscle, liver, heart, and pancreas. However, excessive amounts of FFAs adversely affect health. Plasma FFA levels increase from 200 to 400 μΜ at a basal level up to 800–1500 μΜ in patients with obesity, type 2 diabetes mellitus [29] and hyperthyroidism [6]. Concentrations of 100–400 μΜ, at which palmitate significantly induces IL-6 and MCP-1 secretion in orbital fibroblasts, would be pathologically relevant in patients with hyperthyroidism, as the proportion of palmitate in FFAs is about 25% [30] and plasma FFAs levels increase to 800–1,000 μΜ in patients with hyperthyroidism [6].

FFA exists as albumin-bound and unbound forms. Although 99% or more of FFAs are bound to albumin, the small fraction of unbound FFA is the physiologically more active form [31]. In the current study, we directly added palmitate-dissolved in NaOH/Et-OH to the culture media to reduce the effect of endotoxin. We dissolved palmitate in NaOH/Et-OH rather than conjugating palmitate with BSA. Schwartz et al. [17] raised the problem of endotoxin contamination even after using a low-endotoxin preparation of BSA while preparing FFA/BSA complexes. In that report, the authors recommended a new method for preparing FFA that we used in this study. In our pilot study, we also detected endotoxin contamination in palmitate-bound to BSA using the ToxinSensor^TM^ Endotoxin Detection System (GenScript, Piscataway, NJ; data not shown). Consistent with this result, treatment with palmitate-bound to BSA significantly increased TNF-α production up to 40 fold higher than that of palmitate-dissolved in NaOH/Et-OH (data not shown). Furthermore, we found that palmitate-dissolved in NaOH/Et-OH was less toxic to RAW 264.7 cells than palmitate-bound to BSA in our pilot study (data not shown). Although, it is uncertain what proportion of palmitate is bound to serum albumin or left in an unbound state when palmitate is directly added to culture media, we used palmitate-dissolved in NaOH/Et-OH instead of palmitate-bound to BSA, as endotoxin could affect palmitate.

There may be limitations to generalizing our results in that TAO not only occurs in hyperthyroid patients but also occurs in hypothyroid and euthyroid patients. Clinical studies are needed to define whether or how palmitate contributes to the development of TAO. However, our results demonstrate the possible involvement of palmitate in promoting inflammation within orbital tissues and, subsequently, in the development of TAO.
